# Predicting athletic performance in track and field athletes based on wearable physiological and psychological indicators: an interpretable machine learning study

**DOI:** 10.3389/fphys.2026.1912453

**Published:** 2026-08-14

**Authors:** Pengfei Zhang

**Affiliations:** Faculty of Education, Languages, Psychology and Music, SEGi University, Kuala Lumpur, Malaysia

**Keywords:** athletic performance, heart rate variability, interpretable machine learning, psychological readiness, SHAP, track and field, training load, wearable sensors

## Abstract

Track-and-field performance fluctuates week to week under the joint influence of training load, recovery, autonomic regulation, and psychological readiness, and wearables now enable continuous physiological monitoring; however, whether bodily signals and readiness markers contribute differently to performance prediction across technical and physical events remains unclear. In a prospective longitudinal study, 132 competitive track-and-field athletes (71 physical-event, 61 technical-event) were monitored over a planned 14-week athlete-week design. One-week lagged morning LnRMSSD, resting heart rate, sleep duration and quality, session-RPE load, cognitive anxiety, and self-efficacy were used to predict subsequent standardized weekly performance change. Extreme gradient boosting (XGBoost) models were evaluated with athlete-level grouped cross-validation. SHapley Additive exPlanations (SHAP) summarized feature contributions, and OLS interaction models with athlete-clustered robust standard errors tested moderation by event type. The integrated XGBoost model showed the highest overall prediction among the evaluated feature sets (mean outer-fold R² = 0.510, RMSE = 0.505, MAE = 0.406; within-athlete centered sensitivity R² = 0.177–0.306), and readiness measures added a consistent increment beyond monitoring-only features (mean ΔR² = 0.088). SHAP identified the ordinal competitive-level code as the largest single contribution (23.4%), followed by lagged physiological and self-efficacy features. Interaction analyses gave partial evidence of event-sensitive moderation, mainly for psychological readiness in technical events, whereas aggregated physiological and load interactions were non-significant. Sensitivity analyses showed that predictive performance decreased after removing the competitive-level code (R² = 0.366) and in within-athlete centered models, indicating that the headline R² reflected both stable athlete-level stratification and week-to-week monitoring signals. Overall, integrated wearable physiological and psychological monitoring can help predict and characterize week-to-week performance change in track-and-field athletes, with the most consistent event-sensitive signal involving psychological readiness, particularly self-efficacy, in technical events, while not implying causal mechanisms.

## 1 Introduction

Monitoring an athlete’s status during training provides essential insight into fatigue, recovery, and the adaptation that ultimately determines competitive performance, and load monitoring has been proposed as a central framework for understanding this training response (Halson, 2014; Bourdon et al., 2017; Impellizzeri et al., 2019). Within track and field, however, performance is not a single physiological construct. Sprinting, middle- and long-distance running, jumping, and throwing depend on distinct biomechanical and biological determinants (Zaras et al., 2021; Valamatos et al., 2022; Baker et al., 2025), so the factors that drive week-to-week change are unlikely to be identical across events. Weekly results vary with accumulated training stimuli, incomplete recovery, and shifting psychological states, and training practice therefore needs monitoring tools that can identify, in advance, athletes at risk of decline or primed for improvement (Halson, 2014; Martins et al., 2021).

The rapid development of big data, the Internet of Things, and artificial intelligence has exposed the sports industry to a wave of digital monitoring technologies, and wearable devices built on machine learning algorithms are now growing rapidly in athlete monitoring (Martins et al., 2021; Ometov et al., 2021; Liu and Zhang, 2022). Wearable-derived indicators such as heart rate variability (HRV), resting heart rate, and sleep-related variables provide repeated, minimally invasive markers of autonomic regulation and recovery status (Esco et al., 2025; Bellenger et al., 2016; Nuuttila et al., 2022; Chiang and Khosla, 2023; Le et al., 2024). Nocturnal and morning HRV in particular has been used to track autonomic recovery and individual responses to training load (Bellenger et al., 2016; Nuuttila et al., 2022), while sleep duration and quality are central to recovery and performance readiness (Walsh et al., 2021; Gong et al., 2024; Kong et al., 2025). Internal training load, commonly quantified as session-RPE load, complements these objective signals (Impellizzeri et al., 2019; Inoue et al., 2022). Importantly, subjective and self-reported measures should not be treated as inferior: systematic evidence indicates that self-reported wellbeing and fatigue can be more sensitive to training response than several commonly used objective measures (Saw et al., 2016; Kósa et al., 2023).

Beyond physiological recovery, psychological readiness is a theoretically relevant but frequently neglected determinant of performance. Cognitive anxiety and self-efficacy are associated with confidence, attentional control, and execution under pressure; anxiety and self-confidence have meta-analytic links with sport performance (Woodman and Hardy, 2003; Craft et al., 2003). For self-efficacy, classic evidence established its association with sport performance (Moritz et al., 2000), and later reviews extended this support across sport-performance contexts (Lochbaum et al., 2023; Aizava et al., 2024; López-Rodríguez et al., 2025). Mental fatigue has likewise been shown to impair sport-specific motor performance (Yuan et al., 2023). Because no single measure can adequately explain week-to-week change, multimodal integration of physiological, load, sleep, and psychological information is more consistent with the reality of training monitoring than any unidimensional model.

Psychological readiness was defined in the present study as a pre-performance cognitive-affective and confidence-related state relevant to execution under training or testing demands. It was represented by two indicators: cognitive anxiety, reflecting worry and threat-related cognitive interference (Woodman and Hardy, 2003; Craft et al., 2003), and self-efficacy, reflecting perceived capability and confidence for sport-specific execution (Moritz et al., 2000; Lochbaum et al., 2023). Together, these two indicators provide complementary coverage of the pre-performance readiness construct, addressing both attentional interference and competence-appraisal pathways, without introducing excessive questionnaire burden within the weekly monitoring protocol.

Machine learning has increasingly been applied in sport science, including in track and field, but many applications remain limited by concerns over validation, feature quality, transparency, and generalizability (Iatropoulos et al., 2025; Claudino et al., 2019; Van Eetvelde et al., 2021). Systematic reviews of injury-risk and performance models repeatedly highlight small samples, optimistic performance estimates, and a high risk of bias, particularly when information leaks between training and testing data (Claudino et al., 2019; Van Eetvelde et al., 2021). To reduce optimistic estimates, model development should adopt transparent reporting and rigorous validation procedures, as recommended by contemporary machine-learning reporting frameworks (Walsh et al., 2021; Collins et al., 2024; Vasey et al., 2021). Gradient-boosted tree methods such as extreme gradient boosting (XGBoost) are well suited to the nonlinear, interacting relationships expected among monitoring variables (Chen and Guestrin, 2016), and SHapley Additive exPlanations (SHAP) provide a principled *post-hoc* means of summarizing feature contributions (Lundberg and Lee, 2017). Such explanations, however, describe model-identified associations rather than causal effects and should not substitute for transparent model reporting (Rudin, 2019).

Two gaps motivate the present study. First, although multi-source fusion has repeatedly been shown to improve performance prediction, few studies have examined whether the prediction pattern of physiological and psychological measures differs systematically across track-and-field event types. Technical events such as jumps and throws may be more sensitive to attentional control, stable motor execution, and psychological readiness (Yuan et al., 2023; Gose and Abraham, 2021; Luan and Mirifar, 2021), whereas physical events such as sprints and distance running may be more closely tied to training load, autonomic recovery, and sleep (Halson, 2014; Bellenger et al., 2016; Walsh et al., 2021). Second, most physiological–psychological fusion studies are cross-sectional, whereas longitudinal monitoring is better suited to capturing the dynamic nature of training response and week-to-week performance change (Halson, 2014; Saw et al., 2016). For practitioners, one-week-ahead performance-change prediction can translate multimodal monitoring into an early prompt for reviewing training-load prescription, recovery planning, and readiness assessment before the subsequent test or competition. The present study addresses both gaps using a prospective athlete-week design in which physiological, load, sleep, and psychological information from a preceding period predicts subsequent standardized performance change, and interpretable machine learning summarizes feature contributions.

**Figure 1:**
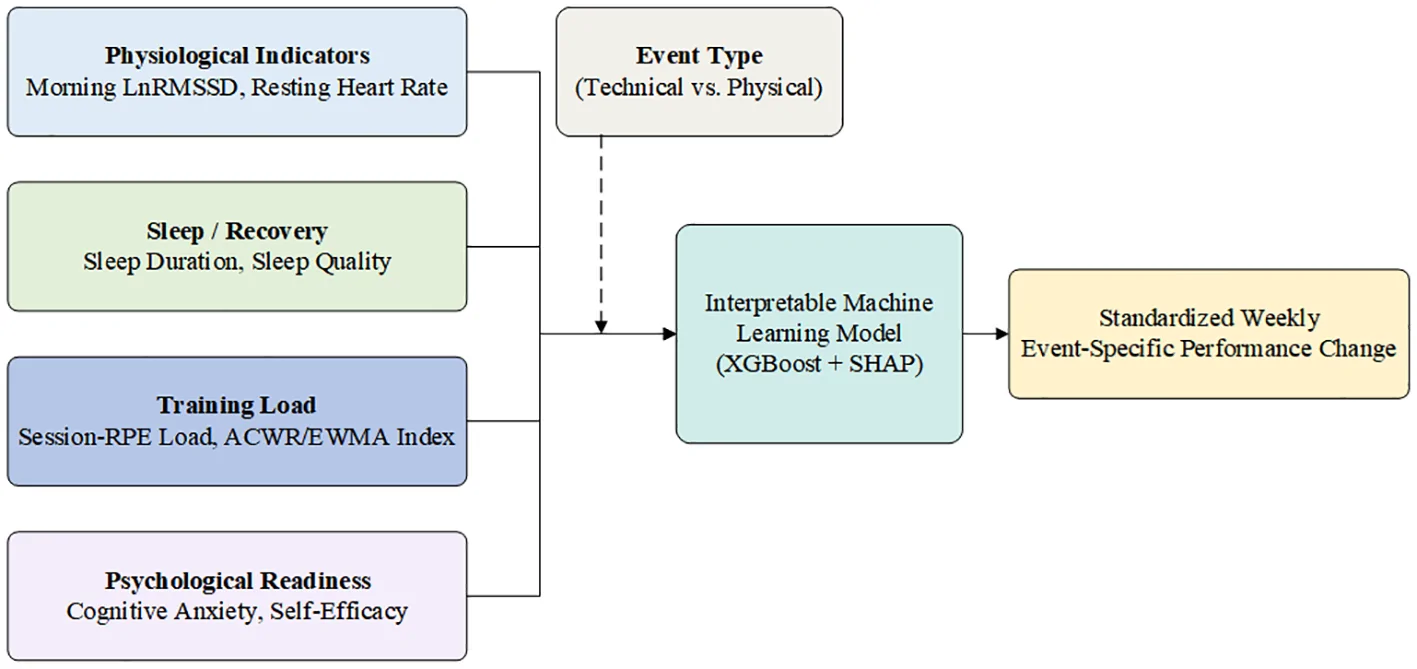
Conceptual framework of the study, linking wearable physiological indicators, sleep/recovery indicators, training-load indicators, and psychological readiness markers to subsequent standardized weekly event-specific performance fluctuation, with event type (technical versus physical) moderating the relative contribution of physiological and psychological domains.

Accordingly, this study had three aims. The first aim (Q1; primary prediction objective) was to determine whether wearable physiological, training-load, sleep/recovery, and psychological-readiness indicators predict subsequent standardized weekly performance change (H1). The second aim (Q2; incremental-value objective) was to test whether cognitive anxiety and self-efficacy provide incremental predictive value beyond physiological, training-load, and sleep/recovery information, such that the full multimodal model outperforms reduced feature-set models (H2). The third aim (Q3; moderation objective) was to estimate whether event type moderated the prediction pattern, with the expectation that psychological-readiness indicators would show stronger associations in technical events whereas physiological and load-related indicators would show stronger associations in physical events (H3). Because event type was a between-athlete moderator, this hypothesis was interpreted with attention to cluster-level precision. SHAP-based feature-ranking, domain-level contribution patterns, auxiliary classification analyses, and sensitivity checks were treated as exploratory or explanatory analyses. The conceptual framework linking wearable physiological indicators, psychological factors, event type, and performance change is shown in **Figure 1**.

## 2 Materials and methods

### 2.1 Study design and participants

This study used a prospective longitudinal observational design to examine whether wearable-derived physiological indicators, training-load measures, sleep-related recovery indicators, and psychological readiness markers predicted subsequent weekly performance change in competitive track-and-field athletes (Halson, 2014; Saw et al., 2016). The study used a rolling-enrollment design across the overall monitoring window from June 3, 2024 to October 26, 2025. Each athlete was scheduled for a planned 14-week monitoring period. The design followed an athlete-week structure, in which monitoring variables from the preceding period were used to predict subsequent standardized event-specific performance variation. The athlete-week design was selected to model within-athlete variation in training response while adjusting for stable between-athlete differences that could affect athlete-week predictions.

A total of 154 track-and-field athletes were screened, of whom 148 met the eligibility criteria and 138 were enrolled in the monitoring program. Six athletes were excluded at the participant level before the construction of lagged predictor-outcome pairs because the participant tracking log recorded early withdrawal or incomplete longitudinal follow-up before final-analysis candidacy was locked (withdrawal weeks 5-12); these athletes had raw monitoring summaries but contributed no eligible lagged pairs to the locked modeling files. The final analysis sample consisted of 132 competitive track-and-field athletes (71 physical-event, 61 technical-event; 83 men, 49 women; mean age = 20.54 years, SD = 1.63; mean training history = 5.93 years, SD = 1.63; competitive-level distribution: national level 2 = 74, national level 1 = 46, elite reserve = 12).

**Figure 2:**
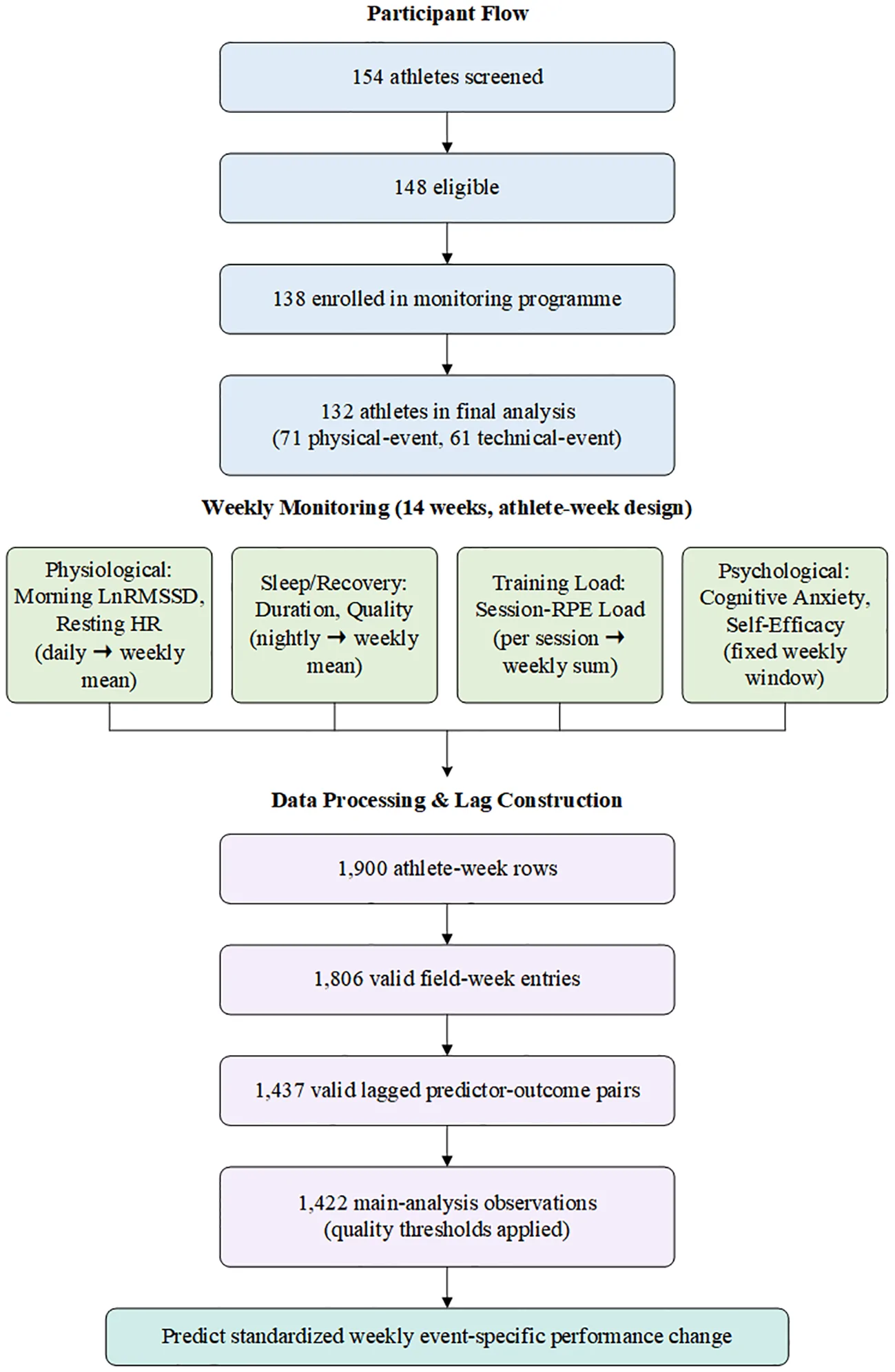
Study design and weekly monitoring workflow showing the prospective longitudinal athlete-week structure across the planned 14-week period, in which lagged physiological, sleep, training-load, and psychological information was used to predict subsequent standardized event-specific performance fluctuation.

Participants were competitive track-and-field athletes aged 18 years or older who were engaged in regular training during the monitoring period. Participant-level final-analysis candidacy was distinct from athlete-week analysis-readiness thresholds: the six non-candidate athletes were not counted in the 1,437 lagged predictor-outcome pairs, whereas subsequent row-level exclusions were applied within the 132 final-analysis candidates. Athlete-week exclusions were then based on missing key outcome data, abnormal-week flags that invalidated the target weekly observation, or records that prevented construction of valid lagged predictor-outcome pairs. The study design and weekly monitoring workflow are summarized in **Figure 2**. Data collection was coordinated by the author and conducted according to the prespecified monitoring workflow with assistance from on-site training staff and coaches. Ethical approval was obtained from the SEGi Research Ethics Committee (Ethics Approval Number: SEGiEC/SR/FOELPM/163/2024-2025). The study was conducted in accordance with the principles of the Declaration of Helsinki. Written informed consent was obtained from all participants, and anonymized data were handled in accordance with institutional privacy requirements.

### 2.2 Monitoring measures

Monitoring variables were organized into four domains: physiological indicators, training load, sleep/recovery, and psychological readiness. **Table 1** summarizes the variables, source fields, timing, collection frequency, calculation rules, and preprocessing rules.

**Table 1:** Monitoring variables, source fields, timing, collection frequency, calculation rules, and preprocessing rules.

Domain	Variable	Source/instrument	Timing & frequency	Calculation	Preprocessing
Physiological	Morning LnRMSSD	Polar H10 chest strap/Polar Verity Sense optical armband; Polar Flow + Kubios HRV Standard 3.5	Morning, daily → weekly mean	ln(RMSSD)	Validity flag and artifact check; daily → weekly mean
Physiological	Resting heart rate	Polar H10 chest strap/Polar Verity Sense optical armband; Polar Flow + Kubios HRV Standard 3.5	Morning, daily → weekly mean	Mean resting HR (bpm)	Validity flag; daily → weekly mean
Sleep/recovery	Sleep duration	Amazfit GTR 4, Garmin Forerunner 255, or Huawei Watch GT 3; app export + user sleep log	Nightly, daily → weekly mean	Hours per night	Sleep-record flag; daily → weekly mean
Sleep/recovery	Sleep quality	Amazfit GTR 4, Garmin Forerunner 255, or Huawei Watch GT 3; app export + user sleep log	Nightly, daily → weekly mean	Quality score	Sleep-record flag; daily → weekly mean
Training load	Session-RPE load	Training log + 0–10 RPE	Per session → weekly sum	Duration × RPE (AU)	Completed-session flag; weekly aggregation
Training load	Acute load (7-day)	Analysis-wide load table	Rolling 7-day	Rolling recent load (AU)	Derived from weekly session-RPE load
Training load	Chronic load (28-day)	Analysis-wide load table	Rolling 28-day	Rolling chronic load (AU)	Derived from weekly session-RPE load
Training load	EWMA-derived workload-ratio index	Derived	Weekly	EWMA-derived workload-ratio index	Derived feature only; not primary load construct
Psychological	Cognitive anxiety	CSAI-2R cognitive anxiety subscale short form; Wenjuanxing/REDCap/paper	Fixed weekly pre-test window	7-item sum when ≥6/7 items present	Attention/completion checks; higher score = more worry
Psychological	Self-efficacy	Sport-specific Self-Efficacy Short Form; Wenjuanxing/REDCap/paper	Fixed weekly pre-test window	8-item sum when ≥7/8 items present	Attention/completion checks; higher score = greater confidence

AU, arbitrary units; ACWR, acute-to-chronic workload ratio; EWMA, exponentially weighted moving average; HR, heart rate; HRV, heart rate variability; LnRMSSD, natural logarithm of the root mean square of successive RR-interval differences; RPE, rating of perceived exertion. All predictors used in modeling were constructed from information available before the outcome week.

Physiological indicators consisted of morning LnRMSSD and resting heart rate. Morning HRV was recorded before breakfast in a resting position using Polar H10 chest straps (ECG chest strap; 1,000 Hz) or Polar Verity Sense optical armbands (PPG optical armband; 135 Hz), exported through the Polar Flow mobile app, and processed in Kubios HRV Standard 3.5. Daily valid records were aggregated to weekly means. LnRMSSD was treated as an autonomic recovery-related marker, and resting heart rate was treated as a complementary indicator of physiological strain and recovery status (Esco et al., 2025; Bellenger et al., 2016; Nuuttila et al., 2022). HRV records were retained when the validity flag was passed and artifact percentage had been checked, including device-auto, Kubios-auto, and manual-review artifact flags. Of the 132 athletes in the main analysis, 112 used Polar H10 chest straps (ECG; 1,220 athlete-week rows in the main lagged matrix) and 20 used Polar Verity Sense optical armbands (PPG; 202 rows). Sensor type was fixed at the athlete level throughout monitoring. We did not establish measurement-level equivalence between ECG- and PPG-derived HRV in this cohort; instead, sensor type was documented, uniform artifact and validity rules were applied before weekly aggregation, and device-sensitivity analyses were used to examine whether the substantive predictive results were dependent on sensor type. Caffeine intake, alcohol use, medication changes during the monitoring period, and menstrual-cycle phase were not systematically recorded prior to HRV measurement.

Sleep/recovery indicators included sleep duration and sleep quality. Sleep was recorded using non-dominant-wrist wearable devices (Amazfit GTR 4, Garmin Forerunner 255, or Huawei Watch GT 3) and exported through the corresponding Zepp App, Garmin Connect, or Huawei Health platform; the sleep variables reflected the wearable sleep algorithm supplemented by user sleep-log review when required. These variables were collected daily at night or the following morning and aggregated to weekly means, because sleep is central to athlete recovery and performance readiness (Walsh et al., 2021; Gong et al., 2024; Kong et al., 2025; Chiang and Khosla, 2023; Le et al., 2024). Valid sleep records were retained according to the predefined sleep-record flag. Daily synchronization reminders were used, and weekly upload summaries were checked for completeness. Sleep records were screened for manual-edit flags, watch-removal periods, and low-battery nights.

Training-load indicators included weekly session-RPE load, the corresponding 7-day acute-load measure, 28-day chronic load, and an acute-to-chronic workload ratio (ACWR) derived using an exponentially weighted moving average (EWMA). Session-RPE load was calculated as training duration multiplied by the 0–10 RPE score and was retained for completed training sessions (Impellizzeri et al., 2019; Inoue et al., 2022). Because the athlete-week data structure aggregated training load over the same 7-day window, weekly session-RPE load and 7-day acute load represented the same weekly load construct in the present analysis. Chronic load indicators were derived from the analysis-wide table according to rolling chronic-load rules. The EWMA-derived workload-ratio index was treated as a derived indicator only and not as the primary training-load construct (Seco-Serna et al., 2024). Because weekly session-RPE total load and 7-day acute load are derived from the same underlying training-session data, these two fields should not be interpreted as independent training-load signals in the current athlete-week aggregation design.

Psychological readiness was represented by cognitive anxiety and self-efficacy. Cognitive anxiety was measured with a CSAI-2R cognitive anxiety subscale short form anchored to the Competitive State Anxiety Inventory-2 Revised cognitive anxiety subscale (Cox et al., 2003), using a response anchor from 1 = not at all to 4 = very much so. Self-efficacy was measured with a sport-specific self-efficacy short form constructed according to Bandura’s self-efficacy scale-construction guidance and sport self-efficacy literature (Bandura, 2006; Moritz et al., 2000), using a response anchor from 1 = not confident at all to 5 = highly confident. Responses were collected through Wenjuanxing, REDCap offline import, or later-entered paper forms in a fixed weekly pre-test window (mean 30.7 min before performance testing, range 10–86 min). Cognitive anxiety and self-efficacy scores were calculated as item sums when at least 6 of 7 and 7 of 8 items were present, respectively. Records were retained when attention checks and completion flags were passed, and duplicate-submission tracking was incorporated into the questionnaire protocol. Cognitive anxiety was included because of its established meta-analytic relationship with sport performance (Woodman and Hardy, 2003; Craft et al., 2003), and self-efficacy because recent reviews support its relevance across sport-performance settings (Lochbaum et al., 2023; Aizava et al., 2024). These two psychological indicators were selected to keep athlete burden low and to focus on psychological readiness constructs directly relevant to pressure-related execution and confidence (Saw et al., 2016; Yuan et al., 2023). Assessment timing was recorded descriptively but was not modelled as a separate predictor in the primary analysis.

Compliance and data quality. Observed adherence was as follows: athletes provided a mean of 5.49 valid HRV days per week, 5.62 valid sleep nights per week, and 4.50 completed training-session RPE entries per week, with a mean data-upload delay of 0.74 days. Device calibration checks were documented in 72.7% of athlete-week compliance records. HRV validity was assessed using a prespecified threshold of artifact percentage not exceeding 8%, plus exclusion of illness-morning, movement, and short-recording flags. Session-RPE was recorded 15–30 min after training and checked against coach or collector logs.

### 2.3 Performance outcome

Weekly performance tests were conducted at the same venue using standardized warm-up procedures. A primary tester conducted testing when available, with a backup tester supervising or replacing the primary tester when required. Missed performance tests due to injury, illness, or schedule conflict were documented and not retrospectively replaced by retesting.

The primary outcome was standardized weekly event-specific performance fluctuation. Weekly performance was based on the athlete’s primary event or a highly related event-specific performance test and was standardized within event-specific testing contexts rather than treated as equivalent to official competition performance (Zaras et al., 2021; Valamatos et al., 2022; Baker et al., 2025). Raw performance was converted to performance percent baseline, and weekly fluctuation was derived as the change from the athlete-specific baseline or reference value. The outcome was expressed in percentage points.

Repeat testing was used to estimate event/test-specific typical error, which was used to distinguish interpretable performance fluctuation from measurement noise in auxiliary analyses (Hopkins, 2000; Atkinson and Nevill, 1998). The primary regression outcome remained continuous performance fluctuation, rather than a binary or three-class change category, because this retained more information about weekly variation.

### 2.4 Data preprocessing and feature engineering

A lagged athlete-week was considered analysis-ready when the predictor week contained at least three valid HRV days, at least one valid sleep night, at least two completed training sessions, and a valid subsequent performance outcome after quality control. Of 1,437 lagged predictor-outcome pairs constructed before applying these thresholds, 1,422 met the criteria and constituted the main analysis matrix. Across the monitoring records, athlete-weeks with logged injury, illness, equipment-failure, or schedule-conflict events were flagged as abnormal weeks; an abnormal week was excluded only when the logged event invalidated the outcome or prevented construction of a valid lagged predictor-outcome pair. In total, 44 athlete-weeks were flagged as outcome-invalidating abnormal weeks during preprocessing (32 injury-related, 12 illness-related). Domain-level missingness was handled through the analysis-readiness thresholds rather than through the abnormal-week flag.

The planned monitoring period consisted of 14 weeks. Raw monitoring files across all 138 enrolled athletes, including the six athletes later excluded at the participant level, produced 1,900 athlete-week rows, of which 1,806 had valid field-week entries. In parallel, performance outcome data were available for 1,844 athlete-week records before lag construction and analysis-specific filtering. Lagged predictor-outcome construction then produced 1,437 valid lagged predictor-outcome pairs. After applying the prespecified main-analysis quality thresholds (at least three valid HRV days, one valid sleep night, and two valid training sessions in the relevant week), the primary grouped prediction, domain-ablation, SHAP, and interaction analyses used 1,422 observations from the same 132 athletes. Sensitivity analyses used analysis-specific variants of this same analysis matrix.

Missingness was summarized at the variable and event-group levels. Complete-case matrices were constructed for each analysis according to the variables required by that analysis. No imputation was used for the primary XGBoost analyses because the 1,422-row main matrix had complete values for all required lagged predictors and the outcome after the analysis-readiness thresholds were applied. Preprocessing, scaling, and feature engineering steps were kept within the training data of the cross-validation workflow to reduce information leakage.

### 2.5 Machine learning, validation, and interpretation

The primary modeling task was regression prediction of standardized weekly event-specific performance fluctuation. Four feature sets were evaluated: a baseline model, a non-psychological monitoring model (hereafter, monitoring-only model), a psychological model, and a combined model. The baseline model included athlete and event-level covariates. The monitoring-only model included wearable physiological, training-load, and sleep/recovery variables. The psychological model included cognitive anxiety and self-efficacy indicators. The combined model included all baseline, physiological, training-load, sleep/recovery, and psychological indicators.

XGBoost was selected as the main nonlinear estimator because gradient-boosted trees can model nonlinear and interacting predictors while allowing explicit regularization (Chen and Guestrin, 2016). The feature set was limited to 17 prespecified raw predictors (19 encoded columns after one-hot encoding of sex and event group) organized into conceptually grounded predictor groups. The model-fitting matrix contained 1,422 athlete-week rows from 132 athlete clusters. Models were evaluated with five-fold athlete-level grouped cross-validation so that observations from the same athlete were not split across training and testing sets within the same outer validation fold, reducing leakage from repeated measurements (Walsh et al., 2021; Collins et al., 2024). All hyperparameters were tuned within this grouped cross-validation loop; tuned complexity controls included L2 penalty, constrained tree depth, minimum child weight, row and column subsampling, low learning rate, and a limited number of estimators. Model performance was summarized across five outer folds using R², RMSE, and MAE, with train-test R² gaps reported as an overfitting diagnostic. Hyperparameter information is reported in **Supplementary Table 1**, and the machine-learning reporting checklist is provided in the **Supplementary Materials**.

SHAP values were used to summarize feature contributions in the combined XGBoost model (Lundberg and Lee, 2017). SHAP analyses were reported at two levels: individual feature ranking and domain-level contribution. Domain-level contributions were calculated for baseline covariates, physiology, psychology, sleep/recovery, and training load, both overall and separately for physical and technical events. SHAP values were interpreted as model-identified feature contributions and not as causal effects.

### 2.6 Statistical analysis, ethics, and data availability

The incremental value of psychological indicators was assessed by comparing the combined model with the monitoring-only model, the psychological model, and the baseline model across outer folds. Differences in R², RMSE, and MAE were calculated for each fold, and paired tests were used to describe whether cross-fold performance differences were directionally consistent. Because these paired tests were based on only five outer-fold differences, the corresponding p values were interpreted descriptively as fold-level consistency checks rather than as strong independent inferential evidence.

Event-type moderation was examined with cluster-adjusted interaction models. Each OLS model included one key standardized lagged predictor, event type, their interaction, and covariates; athlete-cluster robust standard errors accounted for within-athlete dependence. The interaction term tested whether the association between a predictor and subsequent performance fluctuation differed between technical and physical events. Aggregated core interaction terms were also reported for psychological readiness, physiological readiness, and training-load fit. Mixed-effects random-intercept models were evaluated as a preliminary specification for the aggregated interaction terms, but the random-effects covariance estimates were singular and did not yield stable fixed-effect inference. The reported interaction results therefore used athlete-cluster robust OLS as the stable inferential specification. These interaction models were interpreted as cluster-adjusted association tests, and SHAP analyses were treated as model-contribution summaries. No conventional *a priori* power analysis was conducted for the machine-learning prediction task; the sample size was determined by the available longitudinal cohort of competitive track-and-field athletes who met the eligibility criteria and entered the planned 14-week monitoring period. For the event-type interaction analyses, *post hoc* cluster-level precision summaries were calculated. The approximate 80% minimum detectable effect (MDE) was calculated as (z0.975 + z0.80) x the cluster-robust standard error, approximately 2.80 x SE, for a two-sided alpha of 0.05 under a large-sample normal approximation. This value is reported as a *post hoc* precision summary.

Sensitivity analyses examined whether the main findings were robust to alternative analytic decisions. These included using a 14-day training-load window, excluding flagged injury or abnormal weeks, removing the ordinal competitive-level covariate, within-athlete centering of predictors and outcomes, adding a lagged sensor-type covariate to assess device sensitivity, and applying time-respecting validation splits. Auxiliary classification analyses were conducted for two secondary outcomes: whether performance change exceeded typical error and whether weekly change represented decline, stability, or improvement. These classification analyses were treated as supportive only; the primary inference was based on the continuous performance-fluctuation outcome.

All procedures were approved by the SEGi Research Ethics Committee (Ethics Approval Number: SEGiEC/SR/FOELPM/163/2024-2025). The study was conducted in accordance with the principles of the Declaration of Helsinki. Written informed consent was obtained from all participants. Participant data were handled in anonymized form for analysis. The de-identified analysis dataset and reproducibility files supporting the main analyses are provided as **Supplementary Data Sheet S1**; additional data may be made available upon reasonable request subject to applicable ethics and privacy requirements.

## 3 Results

### 3.1 Participant characteristics and monitoring completeness

Participant characteristics and monitoring completeness are summarized in **Table 2**. Briefly, the final analysis sample included 132 competitive track-and-field athletes, with both physical and technical event groups represented.

**Table 2:** Participant characteristics and monitoring completeness by event type.

Characteristic	All (n = 132)	Physical (n = 71)	Technical (n = 61)
Men, n (%)	83 (62.9)	47 (66.2)	36 (59.0)
Women, n (%)	49 (37.1)	24 (33.8)	25 (41.0)
Age, years, mean (SD)	20.54 (1.63)	20.44 (1.67)	20.66 (1.58)
Training history, years, mean (SD)	5.93 (1.63)	5.96 (1.64)	5.90 (1.63)
Valid monitoring weeks, mean (SD)	13.29 (0.86)	13.30 (0.85)	13.28 (0.88)
Valid HRV days, mean (SD)	75.44 (7.58)	76.41 (7.52)	74.31 (7.55)
Valid training sessions, mean (SD)	62.74 (4.68)	65.75 (3.48)	59.25 (3.25)
Valid sleep nights, mean (SD)	77.73 (7.46)	78.00 (8.01)	77.41 (6.81)

HRV, heart rate variability; SD, standard deviation. The two event groups were comparable in age, height, weight, training years, competitive level, and ordinal competitive-level code; full descriptive statistics are reported in **Supplementary Table 2**.

The physical-event group and technical-event group were comparable in age, height, weight, training years, competitive level, and ordinal competitive-level code. The physical-event group included 20 athletes in the 100 m, 20 in the 200 m, 20 in the 400 m, and 11 in middle-/long-distance events. The technical-event group included 12 high-jump athletes, 19 long-jump athletes, 14 triple-jump athletes, and 16 throwing-event athletes. Baseline performance value differed between groups because event-specific units and performance scales differed substantially across physical and technical events; therefore, baseline performance was treated as an adjustment variable rather than interpreted as a simple between-group performance comparison.

Monitoring completeness was high across the planned monitoring period, with similar valid monitoring-week counts in physical and technical events. Valid HRV days, training-session records, and sleep nights are reported in **Table 2**; valid training-session counts were higher in physical events, reflecting differences in training-session frequency and event demands.

### 3.2 Descriptive profiles of monitoring indicators and performance fluctuation

Descriptive profiles of physiological, sleep/recovery, training-load, psychological, and performance-fluctuation variables are reported in **Supplementary Table 2**.

Psychological records had higher missingness than daily physiological, sleep, and load indicators, and the final main-analysis matrix was constructed after lagged predictor-outcome pairing and analysis-readiness thresholds. Complete missingness and descriptive statistics are reported in **Supplementary Table 2**.

### 3.3 Predictive performance of reduced feature-set and combined models

Under athlete-level grouped cross-validation, the full feature set showed the highest overall prediction among the evaluated feature sets for weekly performance change (**Table 3**). The baseline model achieved a mean outer-fold R² of 0.223 (SD = 0.030), RMSE of 0.637, and MAE of 0.513. The monitoring-only model improved performance substantially, with a mean R² of 0.423 (SD = 0.061), RMSE of 0.549, and MAE of 0.438. The psychological model performed better than baseline but below this reduced monitoring comparator, with a mean R² of 0.301 (SD = 0.041), RMSE of 0.604, and MAE of 0.486.

**Table 3:** Predictive performance of reduced feature-set and combined models under athlete-level grouped cross-validation.

Model	R², mean (SD)	RMSE	MAE	ΔR² vs. baseline
Baseline	0.223 (0.030)	0.637	0.513	—
Non-psychological monitoring	0.423 (0.061)	0.549	0.438	0.199
Psychological	0.301 (0.041)	0.604	0.486	0.077
Combined	0.510 (0.044)	0.505	0.406	0.287

MAE, mean absolute error; R², coefficient of determination; RMSE, root mean square error; SD, standard deviation. Values are means across five outer folds. The approximate 95% interval for the combined-model R² was 0.472 to 0.549. The mean train–test R² gap for the combined model was 0.177. These grouped prediction models used 1,422 complete-case observations from 132 athletes.

The integrated model achieved a mean R² of 0.510 (SD = 0.044), RMSE of 0.505, and MAE of 0.406 across the five outer folds. Its approximate 95% interval for R² was 0.472 to 0.549. Compared with the baseline model, this model improved R² by 0.287 and reduced RMSE by 0.132 and MAE by 0.107. The mean train–test R² gap was 0.177, suggesting moderate but not extreme overfitting under the grouped validation design. A full decomposition of the R² into stable between-athlete and within-athlete components is reported in Section 3.6.

### 3.4 Incremental value of psychological indicators

**Figure 3:**
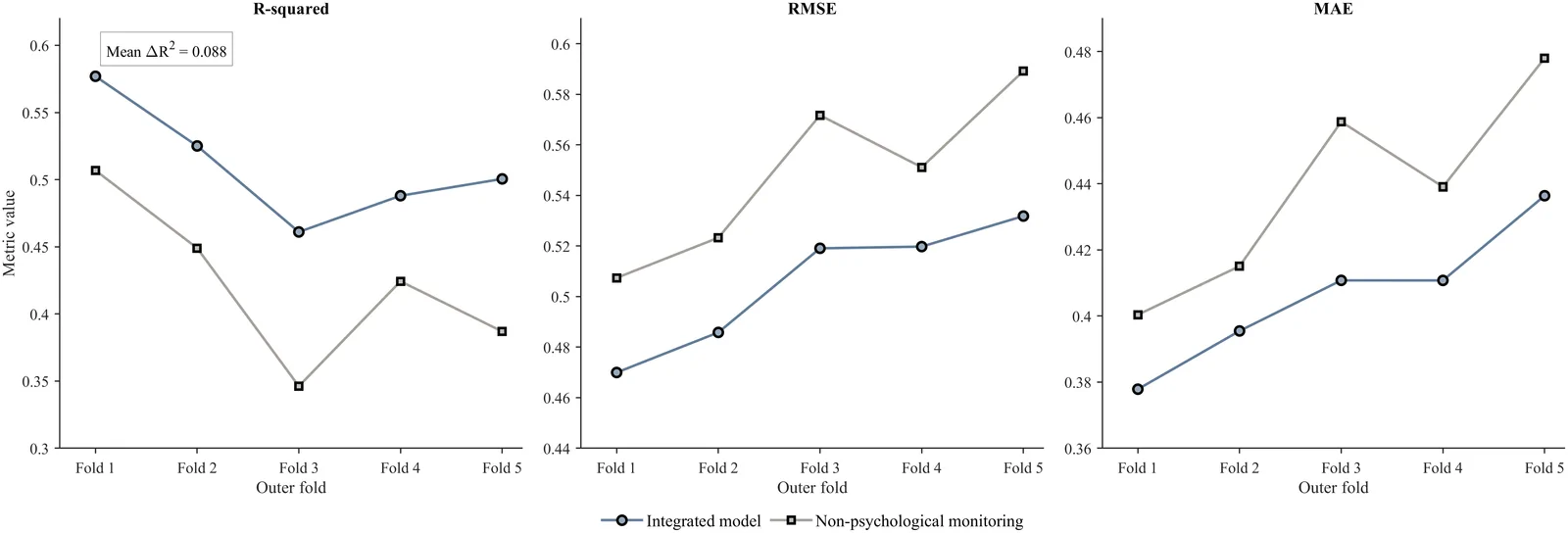
Incremental predictive value of cognitive anxiety and self-efficacy across five outer folds of athlete-level grouped cross-validation, showing the consistent improvement of the integrated model over the monitoring-only comparator in R², RMSE, and MAE.

Adding cognitive anxiety and self-efficacy to the monitoring-only feature set produced a consistent improvement (**Figure 3** and **Table 4**). Although bounded in absolute terms (ΔR² ≈ 0.09), the improvement was directionally consistent across all five folds. Across the five outer folds, the integrated model outperformed the reduced monitoring comparator in R², RMSE, and MAE in all folds. The mean R² improvement was 0.088 (SD = 0.025), with a mean RMSE reduction of 0.043 and a mean MAE reduction of 0.032. The paired fold-level p value for ΔR² was 0.0014, but because it was based on only five outer folds, it was interpreted descriptively as a consistency check rather than as independent inferential evidence. This pattern indicates that cognitive anxiety and self-efficacy added incremental predictive information beyond physiological, load, and sleep/recovery measures.

**Table 4:** Domain ablation and incremental performance analysis.

Model/ablation	R²	RMSE	MAE
Full combined model	0.510	0.505	0.406
Without physiology	0.372	0.573	0.460
Without psychology	0.423	0.549	0.438
Without sleep/recovery	0.479	0.522	0.420
Without training load	0.510	0.505	0.407

MAE, mean absolute error; R², coefficient of determination; RMSE, root mean square error. Incremental comparisons across five outer folds: combined vs. non-psychological monitoring mean ΔR² = 0.088 (paired fold-level p = 0.0014); combined vs. psychological mean ΔR² = 0.210 (fold-level p = 0.0005); combined vs. baseline mean ΔR² = 0.287 (fold-level p < 0.001). These p values are based on five outer-fold differences and are interpreted descriptively as consistency checks. Domain ablations used the same 1,422-observation matrix as **Table 3**, refitting the combined model after removing each monitoring domain while retaining baseline covariates, including the ordinal competitive-level code.

The same model also outperformed the psychological model alone in all five folds, with a mean R² improvement of 0.210 (SD = 0.045), mean RMSE reduction of 0.099, and mean MAE reduction of 0.080; the paired fold-level p value for ΔR² was 0.0005. Compared with the baseline model, it improved R² by 0.287 (SD = 0.038; fold-level p < 0.001). The model without psychological indicators also improved over baseline in all folds (mean ΔR² = 0.199; fold-level p = 0.0017), whereas the psychological model showed a smaller but consistent improvement over baseline (mean ΔR² = 0.077; fold-level p = 0.0002). These p values are reported only as descriptive summaries of five fold-level differences; the main interpretation rests on the direction, magnitude, and consistency of the cross-validated improvements.

Domain-ablation models were rerun on the same 1,422-observation athlete-level grouped cross-validation matrix as **Table 3**, so their values are directly comparable with the primary prediction results. The full model reached an R² of 0.510, RMSE of 0.505, and MAE of 0.406. Removing physiological features produced the largest performance reduction (R² = 0.372; ΔR² = 0.139), followed by removing psychological features (R² = 0.423; ΔR² = 0.088) and sleep/recovery features (R² = 0.479; ΔR² = 0.032). Removing the load domain had little effect (R² = 0.510; ΔR² = 0.001), suggesting that the available load indicators contributed less uniquely after physiological, sleep, psychological, and baseline covariates were included. Because baseline covariates were retained in all domain-ablation models, this matrix should be interpreted as comparing monitoring domains rather than testing the importance of the ordinal competitive-level code.

### 3.5 Feature contributions and event-type moderation

**Figure 4:**
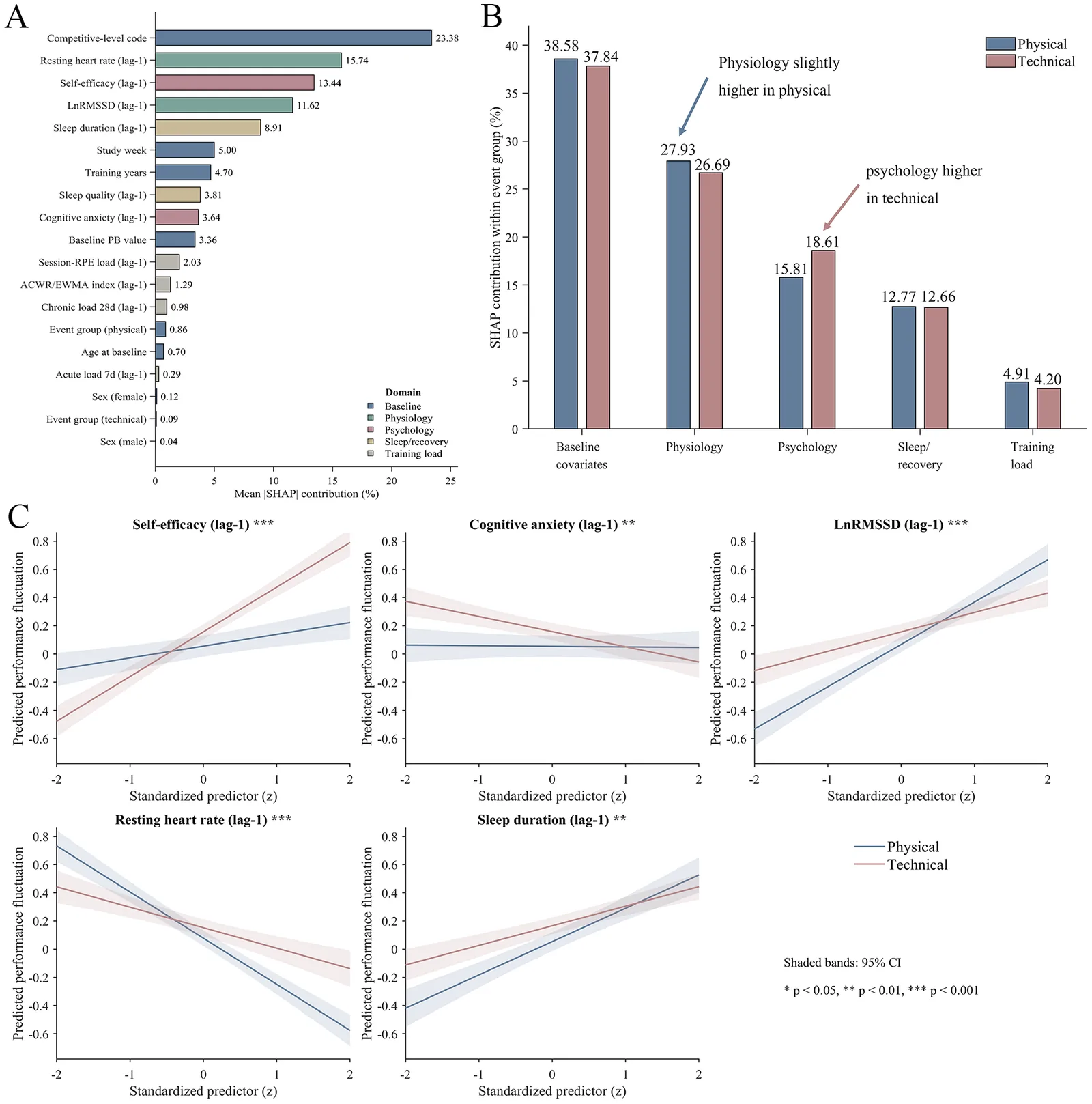
Feature importance and moderation by event type. **(A)** SHAP summary plot for the combined XGBoost model; **(B)** domain-level SHAP contributions stratified by event type; **(C)** event-type interaction effects for key lagged predictors from OLS models with athlete-clustered robust standard errors.

In the combined XGBoost model, the highest-ranked SHAP feature was the ordinal competitive-level code, accounting for 23.4% of mean absolute SHAP contribution (**Figure 4A**). Because this feature was static across the monitoring period, its prominence indicates that part of the prediction reflected stable between-athlete stratification rather than week-to-week monitoring variation alone. Among monitoring variables, the leading features were mean resting heart rate lagged by one week (15.7%), self-efficacy lagged by one week (13.4%), mean LnRMSSD lagged by one week (11.6%), and sleep duration lagged by one week (8.9%). Cognitive anxiety ranked ninth overall (3.6%), sleep quality ranked eighth (3.8%), total weekly session-RPE load ranked eleventh (2.0%), and the EWMA-derived workload-ratio index ranked twelfth (1.3%).

At the domain level, baseline covariates accounted for 38.2% of total absolute SHAP contribution, followed by physiology (27.4%), psychology (17.1%), sleep/recovery (12.7%), and training load (4.6%). When SHAP contributions were stratified by event type, the physiological domain contributed a slightly larger share in physical events than technical events (27.9% vs. 26.7%). In contrast, the psychological domain contributed a larger share in technical events than physical events (18.6% vs. 15.8%). Sleep/recovery and training-load domain contributions were similar across event types, although training-load contribution remained low in both groups (**Figure 4B**).

The interaction analyses provided partial support for the moderation hypothesis (**Figure 4C** and **Table 5**). **Table 5** reports effect sizes, 95% confidence intervals, p values, and approximate 80% minimum detectable effects (MDEs), given the between-athlete nature of event type (132 clusters). In the aggregated summary, only the psychological readiness × technical event interaction had a 95% CI that did not cross zero (β = 0.066, 95% CI: 0.020 to 0.111, approx. 80% MDE = 0.065). The physiological readiness × technical event interaction (β = -0.002, 95% CI: -0.051 to 0.047, approx. 80% MDE = 0.070) and the training-load fit × technical event interaction (β = 0.024, 95% CI: -0.015 to 0.064, approx. 80% MDE = 0.056) had confidence intervals spanning zero. Effects smaller than approximately 0.06-0.07 standardized units would not be estimated with high precision in this 132-cluster design.

**Table 5:** Event-type interaction models testing event-type moderation of key lagged predictors, with athlete-cluster-robust precision summaries.

Interaction term (predictor × technical event)	β	SE	95% CI	p	Approx. 80% MDE
Psychological readiness (aggregated)	0.066	0.023	0.020 to 0.111	0.005	0.065
Physiological readiness (aggregated)	−0.002	0.025	−0.051 to 0.047	0.939	0.070
Training-load fit (aggregated)	0.024	0.020	−0.015 to 0.064	0.222	0.056
Self-efficacy	0.233	0.033	0.169 to 0.298	< 0.001	0.092
Cognitive anxiety	−0.103	0.033	−0.168 to −0.038	0.002	0.093
LnRMSSD	−0.162	0.034	−0.230 to −0.095	< 0.001	0.096
Resting heart rate	0.182	0.037	0.110 to 0.254	< 0.001	0.103
Sleep duration	−0.097	0.036	−0.168 to −0.027	0.007	0.100

CI, confidence interval; LnRMSSD, natural logarithm of the root mean square of successive RR-interval differences; SE, standard error. Coefficients are standardized and derived from OLS models with athlete-cluster robust standard errors; each model included one key standardized lagged predictor, event type, their interaction, and covariates. Positive interaction terms indicate a stronger predictor–outcome association in technical events relative to physical events. Sleep quality, weekly session-RPE load, chronic load, and the EWMA-derived workload-ratio index did not show statistically significant event-type interactions. In the current weekly aggregation, weekly session-RPE load and the 7-day acute-load measure shared the same input values. MixedLM random-intercept models for the aggregated interaction terms produced singular-matrix fitting errors; **Table 5** therefore reports athlete-cluster robust OLS estimates as the stable fallback. These models used 1,422 observations from 132 athletes.

Feature-specific interaction models showed a more differentiated pattern. Among the psychological-readiness indicators, self-efficacy showed the clearest feature-specific technical-event signal. The interaction between self-efficacy and technical-event status was positive (β = 0.233, 95% CI: 0.169 to 0.298, p < 0.001), whereas the interaction between cognitive anxiety and technical-event status was negative (β = −0.103, 95% CI: −0.168 to −0.038, p = 0.002). Selected physiological and recovery-related indicators also showed event-type differences: LnRMSSD had a weaker association in technical events than physical events (interaction β = −0.162, p < 0.001), resting heart rate showed a positive interaction with technical-event status (β = 0.182, p < 0.001), and sleep duration showed a weaker association in technical events (β = −0.097, p = 0.007). These opposing feature-specific directions explain why the aggregated physiological-readiness interaction was close to zero: the null aggregate should not be interpreted as absence of all physiological or recovery-related moderation. Sleep quality, weekly session-RPE load, chronic load, and the EWMA-derived workload-ratio index did not show statistically significant event-type interactions; weekly session-RPE load represented the same 7-day acute-load construct in this aggregation. Because the feature-specific interaction tests involved multiple predictors and were not adjusted for multiplicity, they were interpreted descriptively, with primary emphasis placed on the aggregated domain interactions and precision summaries.

Taken together, these results provide partial support for H3, mainly for psychological readiness in technical events. The precision of interaction estimates is modest given the 132-cluster between-athlete design. SHAP and interaction results suggest that feature-contribution patterns differed between event types, most consistently for psychological readiness and more selectively for physiological and load-related features. However, these patterns should be interpreted with attention to cluster-level precision rather than as confirmatory evidence of systematic moderation.

### 3.6 Robustness and sensitivity analyses

Sensitivity analyses first decomposed the primary performance estimate (**Supplementary Table 3**). The recomputed full combined model yielded R² = 0.513, RMSE = 0.504, and MAE = 0.404, consistent with the primary **Table 3** estimate (R² = 0.510). Removing the competitive-level code reduced R² to 0.366 (RMSE = 0.575, MAE = 0.464), indicating that stable competitive tier contributed materially to the headline predictive performance. Within-athlete centered predictors yielded lower but non-zero predictive performance when predicting the raw outcome (R² = 0.177, RMSE = 0.655, MAE = 0.534) and when predicting the athlete-centered outcome (R² = 0.306, RMSE = 0.477, MAE = 0.377). These results indicate that the headline R² combined stable between-athlete stratification with week-to-week monitoring signals, with a smaller dynamic within-athlete component.

Additional robustness checks addressed temporal structure, load-window choices, and sensor type. Temporal sensitivity analyses showed similar performance under time-respecting splits: rolling-forward validation across six one-week-ahead splits by athlete-specific study week produced mean R² = 0.514, and blocked temporal splits yielded R² = 0.547 when training on weeks 10 or earlier and testing on later weeks and R² = 0.567 when training on weeks 11 or earlier and testing on later weeks. These checks evaluated later-week prediction within the monitored cohort and complemented the primary unseen-athlete grouped-CV estimate. Training-load sensitivity analyses showed that removing the 7-day acute-load variable (R² = 0.512), removing the full training-load domain (R² = 0.510), and using a 14-day training-load window (R² = 0.499) did not materially change the primary performance estimate. Device sensitivity analyses were also consistent with the primary model: adding a lagged sensor-type covariate produced R² = 0.508, and restricting the analysis to ECG-only athlete-weeks produced R² = 0.514 based on 1,220 rows from 112 athletes.

Auxiliary classification analyses showed modest discrimination and were therefore treated as supportive rather than primary. For classifying whether weekly performance change exceeded typical error, the XGBoost binary model achieved a mean macro-F1 of 0.552, balanced accuracy of 0.567, and AUC of 0.593. For the three-class decline/stable/improvement outcome, the model achieved a mean macro-F1 of 0.541, balanced accuracy of 0.513, and AUC of 0.769. These results support retaining the continuous standardized outcome for the primary analyses.

## 4 Discussion

### 4.1 Principal findings

Viewed together, the findings position athlete-week monitoring as an integration problem rather than a search for one dominant signal. Wearable physiological, recovery, load, and psychological data each captured a different part of short-term readiness, but their value depended on how they were combined and on the type of event being monitored. This interpretation is consistent with training-load theory, evidence on subjective monitoring, and recent calls for transparent multi-source modeling in sport science (Halson, 2014; Saw et al., 2016; Martins et al., 2021; Iatropoulos et al., 2025). Importantly, the results do not imply that technical and physical events require separate causal mechanisms; rather, they suggest that the relative usefulness of monitoring domains shifts across event contexts, with limited unique contribution from the available load-derived features after other information was included. This section is organized around the three study aims. Regarding Q1 (prediction), lagged physiological, sleep/recovery, training-load, and psychological-readiness indicators collectively predicted weekly performance change under athlete-level grouped cross-validation, with the headline R² reflecting both stable between-athlete stratification and week-to-week monitoring signals. Regarding Q2 (incremental value), adding cognitive anxiety and self-efficacy improved prediction beyond non-psychological monitoring information (mean ΔR² = 0.088), indicating that psychological readiness contributes predictive information not captured by wearable physiological and training-load data alone. Regarding Q3 (event-type moderation), H3 was partially supported, mainly for psychological readiness in technical events, and the precision of interaction estimates was modest given the 132-cluster between-athlete design.

### 4.2 Physiological and psychological predictors of weekly performance change

The leading physiological features are biologically plausible. HRV- and resting heart-rate-related variables capture autonomic regulation, a process closely tied to training adaptation and recovery status. Nocturnal and morning HRV have been used to track autonomic recovery and individual responses to training load (Esco et al., 2025; Bellenger et al., 2016; Nuuttila et al., 2022), while resting heart rate offers a complementary, low-burden index of physiological strain. Sleep duration fits the same readiness pathway because sleep shapes recovery, perceived exertion, and sporting performance (Walsh et al., 2021; Gong et al., 2024; Kong et al., 2025; Chiang and Khosla, 2023; Le et al., 2024). Session-RPE and related internal-load measures remain useful because subjective responses may capture aspects of training response not fully reflected by objective signals (Inoue et al., 2022; Saw et al., 2016; Kósa et al., 2023).

Psychological readiness adds a different layer of information. Cognitive anxiety and self-confidence have repeatedly been linked with sport performance (Woodman and Hardy, 2003; Craft et al., 2003), whereas self-efficacy has long-standing meta-analytic support as a correlate of performance (Moritz et al., 2000). More recent reviews extend this evidence base across sport-performance contexts (Lochbaum et al., 2023; Aizava et al., 2024). In the present model, self-efficacy ranked among the leading monitoring features, whereas cognitive anxiety contributed more modestly. Mental fatigue offers another pathway through which psychological state may shape weekly execution (Yuan et al., 2023). The integrated model’s advantage is therefore not simply a statistical gain; it reflects the practical reality that physiological strain, recovery, load, and confidence capture different dimensions of readiness.

### 4.3 Event-specific moderation in track and field

The event-specific pattern is easiest to understand from the demands of the events themselves. Jumps and throws depend heavily on attentional control, stable motor execution, and confidence; small disruptions in focus may therefore have disproportionate effects on technical outcomes (Yuan et al., 2023; Gose and Abraham, 2021; Luan and Mirifar, 2021). Sprinting and distance running remain physiologically demanding, but in the present data physiological and recovery-related features contributed substantially to prediction in both event groups rather than clearly dominating physical events (Halson, 2014; Bellenger et al., 2016; Walsh et al., 2021). Confidence in the psychological-readiness interpretation is supported by partial alignment between SHAP patterns and OLS interaction models. Within this pattern, self-efficacy showed the clearest feature-specific psychological signal in technical events, consistent with the role of perceived capability in executing high-precision skills. For physiological and recovery-related features, however, the two analyses addressed different aspects of the data: SHAP indicated substantial contribution to prediction, whereas interaction models showed mixed feature-specific directions and no aggregate physiological-readiness moderation.

This interpretation has clear boundaries. SHAP quantifies how features contribute to model predictions, not whether manipulating a feature would change performance, and *post-hoc* explainability should be paired with transparent model reporting rather than treated as mechanism discovery (Rudin, 2019). The observational athlete-week design also precludes causal inference. The event-sensitive framework is therefore best read as a hypothesis-generating account of where physiological versus psychological monitoring may carry the most predictive information across track-and-field disciplines, conditional on baseline athlete differences.

### 4.4 Comparison with previous studies

The study also differs from much of the recent modeling literature in its target and design. Rather than focusing on injury risk or a single sprint-specific or biomarker snapshot, it examined weekly performance change using longitudinal physiological and psychological monitoring (Gao and Chen, 2025; Santhosh and Kaarthick, 2025; Iatropoulos et al., 2025). This matters because systematic reviews of machine learning in sport science have repeatedly emphasized small samples, data leakage, and inappropriate performance measures as persistent threats to validity (Claudino et al., 2019; Van Eetvelde et al., 2021; Kumar et al., 2025; Rico-González et al., 2023; Yuan et al., 2026). Athlete-level grouped validation and transparent reporting were used to address these concerns, and SHAP outputs were restricted to associative interpretation in line with SHAP’s contribution-based formulation (Lundberg and Lee, 2017).

### 4.5 Practical implications

For training practice, the findings support a cautious, event-sensitive monitoring workflow. Coaches can use HRV, resting heart rate, training load, and sleep as routine readiness checks across event groups, and add closer tracking of confidence and anxiety markers for technical-event athletes. Model outputs are best used as prompts for discussion and review of training adjustment, recovery scheduling, or psychological support, not as automatic thresholds or standalone decision rules. Given that within-athlete sensitivity analyses showed lower predictive performance than the full model, this approach is best viewed as supporting readiness review and monitoring discussions rather than making deterministic week-to-week training or selection decisions. More specifically, a monitoring-based prediction workflow could support coaching decisions in several ways: identifying athletes whose predicted performance trajectory suggests reduced readiness or performance decrement, flagging periods when psychological readiness indicators warrant closer attention, and informing individualized taper or recovery adjustments. The event-type moderation pattern further suggests that psychological readiness monitoring may be particularly informative in technical events where attentional control and confidence are critical for execution. However, this study demonstrates predictive associations rather than a validated decision-support tool; prospective intervention studies are needed to test whether monitoring-guided adjustments causally improve performance outcomes.

### 4.6 Strengths, limitations, and future directions

Several design choices strengthen the analysis: the prospective athlete-week structure, an event-specific track-and-field sample, integrated physiological and psychological monitoring, athlete-level grouped validation to reduce leakage from repeated measurements, interpretable machine learning, and a precision-aware event-type moderation analysis.

Several limitations define the scope of inference. The sample may derive from a limited number of training bases, so external generalizability is uncertain and warrants multi-site validation. The observational design cannot establish causality, and weekly standardized testing is not fully equivalent to official competition performance; typical-error estimation helps distinguish meaningful change from noise but does not eliminate this gap (Hopkins, 2000; Atkinson and Nevill, 1998). *Post-hoc* explanations describe model contributions rather than mechanisms (Rudin, 2019), and the prediction task is best read as athlete-week prediction conditioned on baseline tier and lagged monitoring signals. The primary cross-validation estimates generalization to unseen athletes; temporal sensitivity analyses support time-respecting prediction within the monitored cohort and should be followed by external prospective validation. The headline R² combines stable between-athlete stratification with within-athlete monitoring signal, with a smaller dynamic component in centered analyses.

Measurement and data-collection limitations also remain. Psychological measures were self-reported and may be subject to social-desirability and interpretation biases. Wearable-derived variables carry known measurement error and should be interpreted in light of documented validity and reporting limitations (Chiang and Khosla, 2023; Le et al., 2024; Chan et al., 2022). Weekly session-RPE load and the 7-day acute-load measure used the same aggregation window in this athlete-week dataset, so they represent the same weekly load construct; the unique contribution of the training-load domain was low, and alternative load-window configurations were not exhaustively tested. ECG- and PPG-derived HRV measurements are not perfectly interchangeable, and the device-sensitivity analyses support robustness of the predictive conclusions rather than measurement-level equivalence between sensor types. Caffeine intake, alcohol use, medication changes during monitoring, and menstrual-cycle phase were not systematically recorded, leaving possible residual confounding of HRV and readiness measures.

Future work should pursue multi-site external validation, longer season-spanning monitoring, and integration with intervention studies to test whether recovery- and psychology-focused adjustments causally influence performance. Embedding the model within a training-monitoring system for prospective, real-world evaluation, with transparent reporting and external validation, would be a natural next step.

## 5 Conclusion

The present study showed that lagged physiological, sleep/recovery, training-load, and psychological-readiness monitoring indicators collectively predicted weekly performance change (H1), with the headline R² reflecting both stable between-athlete stratification and week-to-week monitoring signals. Adding cognitive anxiety and self-efficacy improved prediction beyond non-psychological monitoring information (H2; mean ΔR² = 0.088). Event-type moderation (H3) was partially supported, mainly for psychological readiness in technical events, with self-efficacy showing the clearest feature-specific technical-event interaction (β = 0.233). The precision of interaction estimates was modest given the 132-cluster between-athlete design. Sensitivity analyses showed that performance decreased after removing competitive level (R² = 0.366) and in within-athlete centered models (R² = 0.177–0.306), indicating that the dynamic week-to-week signal was smaller than the full-model R². These findings position athlete-week monitoring as an integration problem requiring multimodal data, while acknowledging that between-athlete stratification accounts for a meaningful portion of predictive performance.

## Data Availability

The de-identified analysis dataset and supporting reproducibility files are provided as Supplementary Data Sheet S1. Additional data may be made available by the corresponding author upon reasonable request, subject to applicable ethics and privacy requirements.
